# Magnitude and factors associated with upper respiratory tract infection among under-five children in public health institutions of Aksum town, Tigray, Northern Ethiopia: an institutional based cross-sectional study

**DOI:** 10.11604/pamj.2020.36.307.17849

**Published:** 2020-08-19

**Authors:** Teklay Zeru, Hagos Berihu, Gerezgiher Buruh, Haftom Gebrehiwot

**Affiliations:** 1 Department of Nursing, College of Health Sciences, Aksum University, Aksum, Ethiopia,; 2 Department of Nursing, College of Health Sciences, Mekelle University, Mekelle, Ethiopia

**Keywords:** Upper respiratory tract infection, under-five children, Aksum

## Abstract

**Introduction:**

upper respiratory tract infection is a leading cause of morbidity among under-five, particularly in the developing countries. Delays in the identification and treatment of under-fives are among the main contributors to the complication. The aim of this study was to assess the magnitude and to identify factors associated with upper respiratory tract infection among under-five children, in public health institutions of Aksum City, Tigray Region, North Ethiopia, 2016.

**Methods:**

institutional based cross-sectional study was done. Cases were under-five children who had get service. The study participants were selected using Systematic random sampling technique. Data were entered, using Epi-info version 7 and analyzed using SPSS version 22.0. Clinical data from the chart were used to diagnose upper respiratory tract infection types. The binary logistic regression model was used to test the association between dependent and independent variables and multivariable logistic regression was used to identify the associated factors to upper respiratory tract infections.

**Results:**

out of 213 study participants 52.6% identified as having at least one type of upper respiratory tract infection, i.e. sinusitis 22 (10.3%), 37 (17.4%) otitis media, 39 (18.3%) tonsillitis and common cold 83 (39.0%). Multivariable logistic regression analysis shows that rural residence 7.6 [AOR (95%CI) (2.49, 23.58)], civil servant father's children 4.49 [AOR (95%CI) (1.57, 12.83)], non-immunization 6.0 [AOR(95%CI) (1.38, 26.8)], mud house wall 4.58 [AOR (95%CI) (1.74, 12.0)], rental house 5.1 [AOC (95% CI) (1.82, 14.6] and large family size 5.3 [AOC (95%CI) (2.3, 12.1 )], were found to be statistically associated.

**Conclusion:**

socioeconomic, maternal and environmental factors had contributed to the upper respiratory tract infection. Strengthening of the existing disease prevention policy as well as improvement of institutional health service behavior is crucial.

## Introduction

World Health Organization (WHO) has classified diseases of the respiratory system in the International Classification of Diseases (ICD). Respiratory diseases are categorized according to whether they affect the upper or lower part of the respiratory system [1,2]. Upper respiratory tract infections are the most common and frequently occurring infections in the pediatric population [3]. A strong confirmation for the prevention of the disease is rather inadequate, and thus, the patients take preventive measures on the basis of their own experience or preferences. However, an upper respiratory tract infection is referred to as a viral infection causing inflammation and infection in the nose and throat. Upper respiratory tract infection has been regarded as a nonspecific term that is used to describe acute infections involving the nose, Paranasal sinuses, pharynx, larynx, trachea, and bronchi [4,5]. The common physical factors which can contribute to the infection were urban living area, open waste disposal, exposure to cold, same sickness (URTI) in the family, smoking inside the house and irregular intake of fruits by the child [6]. There is a worldwide increase in antibiotic resistance, largely related to inappropriate use of antibiotics; studies suggest that inappropriate use of antibiotics for upper respiratory tract infection (URTIs) adds to the burden according study in India since 2013 [7].

Childhood upper respiratory tract infections also define as the acute inflammation of nasal or pharyngeal mucosa in the absence of other specifically defined respiratory infection [8]. Housing condition like day care attendance is a well-established risk factor for URTIs in preschool children [9,10]. Uncomplicated URTIs account for 25 million visit to family physicians and under-five children experience six to eight upper respiratory tract infections per year in the United States [11]. Upper respiratory tract infections are common and important, although rarely fatal; they are a source of significant morbidity and carry a considerable economic burden. Each year in the US, $2 billion is spent on over-the-counter preparations to relieve cold symptoms, predominantly in children. Children experience 3-8 colds per year and 10-15% has at least 12 per year, usually associated with overcrowding like attendance at day-care centres or nurseries. In infants, onset is more likely to be associated with a high fever, irritability and nasal obstruction affecting feeding and sleep [12]. A yearly acute upper respiratory infection in under -five children is responsible for an estimated 4.1 million deaths worldwide [13]. Under five years represent about 12% of general population in India, large majority of these children live in rural, tribal areas and in urban slums 30% of the mortality rate shows due to URTIs since 2014 [14]. Upper respiratory tract infections are highly prevalent, especially in children between the ages of two and four years. Children less than six months old are relatively protected against community based respiratory tract infections. The frequency of URTIs increases and becomes high during the second year of a child´s life, and may increase again during childbearing years [15].

Under-five children are risky population to get an upper respiratory tract infection due to lack of immune power. The important risk factor associated with respiratory diseases include malnutrition, low birth weight, climatic variations, overcrowding house, air pollution, poor ventilation and lack of environmental sanitation [16]. The preventive measures of respiratory infections includes hygienic practices related to personal and environmental hygiene, appropriate disposal of respiratory secretions, isolation infected patients, maintenance of nutritional status, immunization to be completed as per schedule and special protection of children during weather variations to prevent cold [17]. In addition prevention and controls of URTI through health education, environmental sanitation, good nutrition and avoid overcrowding is one of the component of primary health care listed since 1978; but this problem is still reported from health facilities. Taking into consideration this study is expected contribute on this problem in the study area as well as Tigray region. Therefore the aim of this study was to assess the magnitude and factors associated with upper respiratory tract infection among under-five children in public health institutions of Aksum town, Tigray, Ethiopia.

## Methods

Institutional based cross-sectional study was conducted in Aksum town which is found in the central zone of Tigray located at 1024 km away from Addis Ababa the capital city of Ethiopia as well as 241 km from the capital city of the Tigray region Mekelle since December 2015 up to June 2. The study was carried out among under-five who were attentive to public health institution of the city. The required Sample size was determined using the single population proportion formula. The following assumptions were made during calculating the sample; 95% confidence interval with a 5% margin of error, the expected proportion of upper respiratory tract infection (P) is 30% in Addis Ababa. Monthly estimated total patient flow of health facilities are 480, use correction formula to calculate the final sample size using the following formula, since total population is <10000; nf = 193 By adding 10% Non-Response rate, the total required sample size = 213 The sample size was proportionally distributed to three health institution based on the number of patients. Systematic random sampling technique by dividing the total number of eligible patient (P) by the required sample size(s), the number “K” obtained by dividing P/s=K was used to identify the interval among under-five from the sampling frame. Different K^th^ interval was calculated for each health facility. Finally since the sampling fraction was '2', every 2^th^ children was included in the study and to select the first child from 480/213 =2 were used.

A structured questionnaire was used and all the variables of interest were assessed accordingly. The questionnaire comprises four parts; socio-demographic and socio-economic factors, child factors, checklist from clinical data and environmental factors. Four diploma nurse data collectors and two BSc nurses supervisors were trained for three days. Initially the questionnaire was prepared in English and it translated to the local language Tigrigna and translated back to English by different individuals to ensure understandability, and check for its consistency. The data was collected for about two months. Participants who were not interested to provide information at the time of data collection was consider as non-response. As a whole data was checked for completeness and after coding for each questionnaire and checklist data was entered into Epi-Info version 7 and the data was cleaned for inconsistencies and missing values and important amendment were done. Final data were exported to window based statistical package for social sciences (SPSS) version 22.0 Odds Ratio at 95% Confidence Interval was used to ascertain the association between dependent and independent variables as appropriate. Bivariate regression analysis was run to assess the association between the dependent and independent variable, then variable which have a P-value of <0.3 was entered to multiple logistic regression analysis to see the independent effect of predictor variables over the outcome variables. During the multivariable analysis the cut-off point for level of significance was P-value < 0.05 from the model. The strength of statically association was measured by adjusted odds ratios and 95% confidence intervals. The goodness of fit the final logistic model was tested by using Hosmer and Lemeshow test at a P-value of > 0.05. The multicollinearity was checked using VIF <2.5/tolerance test near to 1. Finally the result was summarized and presented using text, graph, frequency tables and other summary statistics.

**Ethical approval and consent to participate:** ethical clearance were secured from the Mekelle University, College of Health Science Institution Review Board (IRB) with IRB Number 21/03/2016. Since the data is from staff Nurses permission from Tigray Regional health bureau (TRHB) and woreda offices was taken. Furthermore, written consent was taken from TRHB officer and verbal consent was agreed by all participants. However, data was recorded anonymously and confidentially

## Results

**Socio-demographic characteristics:** in this study, a total of 213 participants whose children under-five years aged were interviewed making the response rate 100%. The mean (±SD) age of mothers was 28.85 (±6.66) years ranges from 15 to 46 years. Sixty-five (30.5%) of mothers was in the age range 25-29 years, 178 (83.6%) were orthodox by religion. The majority of mothers 193 (90.6%), were married. Regarding of residence, 166 (77.9%) were from urban, only 47 (22.1%) were from rural. The largest ethnic group was Tigray, 205 (96.2%), followed by Amara 8(3.8%) (Table 1).

**Table 1:** socio-demographic characteristics of participants attending public health institutions, Aksum town, Northern Ethiopia, 2016(n=213)

Variable		Frequency	Percent
Maternal age	15-19	11	5.2
	20-24	48	22.5
	25-29	65	30.5
	30-34	35	16.4
	≥35	54	25.4
Religion	Orthodox	178	83.6
	Muslim	31	14.6
	Others	4	1.8
Marital status	Married	193	90.6
	Single	12	5.6
	Other	8	3.8
Maternal educational level	Illiterate	37	17.4
	Primary school	76	35.7
	Secondary school	58	27.2
	Higher education	42	19.7
Maternal occupation	Housewife	78	36.6
	Civil servant	47	22.1
	Private employee	88	41.3
Husband's Education level	Illiterate	44	20.7
	Primary school	75	35.2
	Secondary school	56	26.3
	Higher education	38	17.8
Husband's occupation	Farmer	112	52.6
	Civil servant	56	26.3
	Private employee	45	21.1
Household monthly income	<726	53	24.9
	727-1200	55	25.8
	1201-2300	57	26.8
	>2300	48	22.5

**Child-related variables:** study subjects were asked about their caretaker of children; one hundred sixty (75.1%) of them had both parents and only 7(3.3%) of them with other guardians. Regarding of their age group 112(52.6%) of them were 6-35 months and 39(18.3%) were less than 6 months old. Two hundred three (95.5%) were delivered in health facilities out of the 171 (80.3%) were normal weight immediately after delivery (Table 2).

**Table 2:** distribution of children's health related variables of under-five aged children attending public health institutions, Aksum town Northern Ethiopia, 2016 (n-213)

Variable		Frequency	Percent
Caretaker	Mother	46	21.6
	Both parents	160	75.1
	Other guardians	7	3.3
Age of Children	<6 months	39	18.3
	6-35months	112	52.6
	35-59 months	62	29.1
Sex of children	Male	86	40.4
	Female	127	59.6
Place of delivery	Health facility	203	95.5
	At home	10	4.7
Weight during delivery	<2500	42	19.7
	2501-4000	171	80.3
Immunization Status of children	Not fully immunized	24	11.3
	Fully immunized	189	88.7

**Figure 1:**
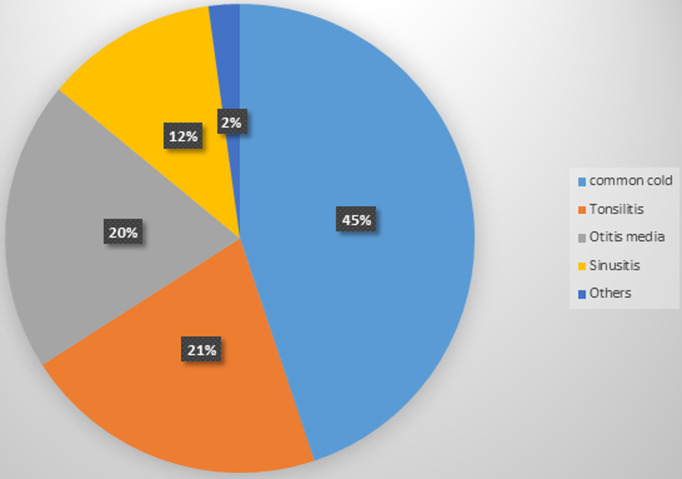
distribution of upper respiratory tract infection types among under-five children who visited public health institutions of Aksum Town, Northern Ethiopia, 2016

**Magnitude and types of upper respiratory tract infections:** this study revealed that from the total respondents, 122 (57.3%) had a history of upper respiratory tract infection, 80 (37.6%) were acquired 1-2 times per year and only 2(0.9%) were above seven times per year. One hundred thirty seven (64.3%) of the children were taken intervention and 78(36.6%) of them were treated by antibiotics. Among the total participants 112 (52.6%) of were presented with at least one type of upper respiratory tract infections. Regarding of upper respiratory tract types rate, sinusitis 22 (10.3%), otitis media 37 (17.4%), tonsillitis 39(18.3%) Figure 1 and common cold 83(39.0%) (Table 3).

**Table 3:** distribution of upper respiratory tract infection types on under-five based on clinical data under-five children attending public health institutions, Aksum town Northern Ethiopia, 2016

Variables		Frequency	Percent
History of upper respiratory tract infection	No	91	42.7
	Yes	122	57.3
Place of visit	heath facility	116	54.5
	Traditional	97	45.5
Occurrence of URTI per year	0	69	32.4
	1-2	80	37.6
	3-4	34	16
	5-6	28	13.1
	≥7	2	0.9
Type of intervention	Antibiotics	78	36.6
	Traditional at home	56	26.3
	Did not take	79	37.1
Currently upper respiratory tract case	No	101	47.4
	Yes	112	52.6
Any type of Malnutrition	No	147	69
	yes	66	31

**Environmental related factors:** all the study subjects were also asked about the living house, 169(79.3%) were non rental house and only 44(20.7%) rental house. One hundred sixty (81.8%) had windows (Table 4).

**Table 4:** distributions of upper respiratory tract infection, environmental related factors among under-five attending in Aksum public health institutions, Aksum town, North Ethiopia, 2016

Variables		Frequency	Percent
House wall type	Mud	150	70.4
	Cement	63	29.6
Availability of windows	No	53	24.9
	Yes	160	75.1
Practice of opening the window	No	56	26.3
	Yes	157	73.7
Mode of transport	public transport	195	91.5
	Traditional ambulance	4	1.9
	Others	14	6.6
Living adequacy	Adequate	151	70.9
	Inadequate	62	29.1
Family size	Small	109	51.2
	Large	104	48.8
Presence of waste disposal	No	60	28.2
	Yes	153	71.8
Disposal system	Take care municipality	108	50.7
	Put on the surrounding area	105	49.3
Frequency Cleaning the room	Once per day Twice per day	193 13	90.6 6.1
	Other	7	3.3

**Factors associated with upper respiratory tract infection:** variables that found significant in the bivariate analysis at a p-value <0.3 were included in the multivariable analysis. Variables in multivariable included were ethnicity, Residence, husband´s occupation, household monthly income, age of children, child´s sex, immunization status, previous URTI, place of visit, history of intervention, type of intervention, house wall type, malnutrition, living condition, house, availability of windows, practice opening window and Family size. Multivariable analysis showed that rural residence was significantly associated with presence of upper respiratory tract infection (P<0.001). The odds of having URTI in rural area is 7.6 compared to urban 7.6 [AOR (95% CI) (2.49, 23.58)]. Civil servant father children´s was statistically associated with upper respiratory tract infection (P<0.05). The odds of having URTI in Civil servant were 4.49 compared farmer 4.49 [AOR (95%CI) (1.57, 12.83)]. Similarly the odds of having URTI in none fully-immunized child is 6 (p<0.01) compared to those who were fully-immunized 6 [AOR (95%CI) (0.43, 2.86)]. The odds of having URTI in mud wall type 4.58 compared with cement 4.58 [AOR (95%CI) (1.74, 12.03)]. Regarding of houses also showed a statistical significant association with upper respiratory tract infection. The odds of having URTI in rental house 5.1 compared to those non rental 5.1 [AOR (95%CI) (1.82, 14.6)]. Family size is significant associated with the upper respiratory tract infection. The odds having URTIs in large family size is 5.3 times higher than family size with small family size compared small family size 5.3 [AOR (95% CI) (2.3, 12.1)]. On the other hand, ethnicity, household monthly income, Age of children, child´s sex, Previous URTI, place of visit, history of intervention, type of intervention, malnutrition, living condition, availability of windows and practice opening window were not statistically associated with upper respiratory tract infection (Table 5).

**Table 5:** associated factors of upper respiratory tract infection among under-five children in public health institutions of Aksum Town, Northern Ethiopia, 2016 (n=213)

Variables		Presence of upper respiratory tract infection			
**No**	**Yes**	**COR[95%C.I]**	**AOR [95%C. I]**
Ethnicity	Tigray	106(48.3%)	99(51.7%)	.35(.07, 1.81)	.28(.04, 1.88)
	Amara	6(25%)	2(75%)	1.0	1.0
Residence	Urban	93(56%)	73(42.9%)	1.0	1.0
	Rural	8(17%)	39(39%)	6.21(2.73, 14.10)***	7.6((2.49, 23.5)***
Husband's occupation	Farmer	56(50%)	56(50%)	1.25(.624, 2.50)	1.0
	Civil servant	36(64.3%)	20(35.7%)	2.25(1.00, 5.02)*	4.4(1.57, 12.8)*
	Private organization	20(44.4%)	25(55.6%)	1.0	2.99(1.07,8.34)
Household monthly income	<726	23(43.4%)	30(56.6%)	1.67(.76, 3.68)	2.15(0.66, 6.9)
	726-1200	24(43.6%)	31(56.4%)	1.66(.76, 3.62)	1.34(0.43, 4.1)
	1201-2300	27(47.4%)	30(52.6%)	1.42(.66, 3.09)	1.35(0.44, 4.18)
	>2300	27(56.3%)	21(43.8%)	1.0	1.0
Age of children	<6 months	22(56.4%)	17(43.6%)	1.0	1.0
	6-35 months	42(37.5%)	70(62.5%	2.15(1.03, 4.51)*	2.49(0.88, 7.0)
	36-59 months	37(56.7%)	25(40.3%)	0.874(.389,1.968)	0.67(0.22,06)
Child sex	Male	37(43%)	49(57%)	1.34(.776, 2.33)	1.11(0.49, 2.5)
	Female	64(50.4)	63(496)	1	1.0
Immunization status	None fully imm.	20(83.3%)	4(16.7%)	5.27(1.73, 16.00)**	6.09(1.38, 26.8)*
	Full immunized	92(48.7%)	97(51.3%)	1.0	1.0
Past URTI history	No	50(54.9%)	41(45.1%)	1.0	1.0
	Yes	51(41.8%)	71(58.2%)	.58(.341, 1.01)***	1.0(0.43, 2.8)
Place of visit	health facility	50(43.1%)	66(56.9%)	.68(.39, 1.17)	0.88(0.36, 2.1)
	Traditional	51(52.6%)	46(47.4%)	1.0	1.0
History of intervention	No	79(57.7%)	58(42.3%)	1.0	1.0
	Yes	22(28.9%)	54(71.1%)	3.34(1.83, 6.09)***	2.41(0.95, 6.4)
Type of intervention	Antibiotics	27(34.6%)	51(65.4%)	2.632(1.38, 5.02)*	2.23(0.82, 6.4)
	Traditional medicine	28(50.0%)	28(50%)	1.39(.700, 2.77)	2.19(0.71, 5.52)
	Didn't use	46(58.2%)	33(41.8%)	1.0	1.0
House wall type	mud	85(56.7%)	65(43.3%)	3.84(2.00, 7.37)***	4.58(1.7, 12.0)**
	cement	16(25%)	47(74.6%)	1.0	1.0
Malnutrition	No	91(54.8%)	75(45.2%)	1.0	1.0
	Yes	10(21.3)	37(78.7%)	4.489(2.09,9.62)***	1.1(.47, 2.6)
Living Condition	Adequate	81(53.6%)	70(46.4%)	1.0	1.0
	Inadequate	20(32.3%)	42(67.7%)	2.43(1.30, 4.52)*	2.2(.81, 6.1)
House	rental	23(76.7%)	7(23.3%)	3.47(1.42 ,8.49)*	5.1(1.8,14.6) **
	Non-rental	89(48.6%)	94(51.4%)	1.0	1.0
Availability of windows	No	22(41.5%)	31(58.5%)	.72(.38, 136)	3.9(0.8, 19.3)
	Yes	79(49.4)	81(50.6%)	1.0	1.0
Opening windows practice	No	23(41.1%)	33(58.9%)	0.706(0.38, 1.30)	0.28(.05, 1.5)
	Yes	78(49.7%)	79(50.3%)	1.0	1.0
Family size	Small	67(61.5%)	42(38.5%)	1.0	1.0
	Large	34(32.1%)	70(67.3%)	3.2(1.8, 5.7)***	5.3(2.3,12.1)***

* p<0.05; **p<0.01; ***p<0.001; **COR**- crude odds ratio; **AOR**- Adjusted odds ratio; **CI**= confidence interval

## Discussion

The purpose of this study was to assess the magnitude and factors associated with upper respiratory tract infection among under-five children in public health institutions of Aksum town. The prevalence of upper respiratory tract infection is clearly recognized and over the years more evident that; it is the most common problem among under-five even in developed countries. In this study, it was found that 52.6% of visited the study participant under-five children were identified as presence of upper respiratory tract infection. This result is higher than a study in Atlanta 24%, (2015) [18]. The difference is due to low economic standard of the population shortage of basic needs like basic sanitation and balance diet. It is also higher than the study conducted In Malaysia 35.8%), 1996 [19]; because of large number of family size and most of the live in rental house. In Uganda 37.4%), (2004) [20]; the difference is due to enough public health professionals create an awareness on prevention and control respiratory infection. But it is less than with the study conducted in Iran Mofid (62%) 2012. The important risk factor associated with respiratory diseases, climatic variations, overcrowding house, air pollution, poor ventilation and lack of environmental sanitation more than Ethiopia; So that, the finding of this study had a good argument back ground and evidence based to find out more or less prevalence rate compared to other studies. In this study the predominant type of respiratory tract infection was common cold (39%), tonsillitis (18.3%), otitis media (17.4%) and sinusitis (10.3%). Although the predominant was common cold; compared with the study conducted in Iraq Mofid this study was relatively smaller since finding were common cold 54%, sinusitis 17.5% but higher than otitis media 8.3% and tonsillitis 5.1% [21]. The possible reason for the predominance could be due to the different species of virus, bacterial infections even normal floral caused infection since immature immunity system of the children.

In this study 80 (37.6%) were infected 1- 2 times per year compared with study conducted in America which was 6-8 in a year [18,22]. This may be the health policy of the country and traditionally most people considered as upper respiratory tract infections are self-limited while in developed country like America give value even mild problem of upper respiratory tract infection needed immediate treatment and follow up. In this study residence was found to be a significant factor of upper respiratory tract infection (p< 0.001). The odds having URTI in rural area 7.6 compared to those urban area 7.6 [AOR (95%CI) (2.49, 23.58)]. This is contradict with study conducted in Indian (2011), the odds of urban residence 2.5 [AOR (95%CI) (1.09, 7.10)] [20, 22]. Likewise, it is congruent with the EDHS result, 2011 [23], which showed mothers who rural residence had a high chance of upper respiratory tract infection than urban residence, In rural area, it is more because of lack of availability of basic health services, lack of awareness, and other associated factors like overcrowding, low socio-economic status, absence of cross ventilation, indoor air pollution were responsible factors. This study showed that civil servant father´s children, the odds of being developed upper respiratory tract infection were 4.49 in civil servant father´s children compared to those farmers 4.49 [AOR (95%CI) (1.57, 12.83)]. But there is no significantly associated in other similar studies. This could be resulted due to difference in exposure to the poor sanitation environment; lack of care taker while in work area may have higher chance to infect. None fully-immunized showed a significant association with upper respiratory tract infection. The present study indicated that the odds of none fully-immunized child 6 times more likely to be suffering from upper respiratory tract infection compared to those fully immunized 6 [AOR (95%CI) (1.38, 26.8)]. This finding is higher compared with the previous finding reported in Atlanta and Uganda [12,24]. This difference might be due to immunization preventable disease causes of upper respiratory tract infection and shows insufficient coverage of EPI service either missed opportunity or refused take vaccine. This is happened because of most URTIs are vaccine preventable, so if they fail to be immunization URTI inevitable.

In this study house wall type had significantly associated; the odds of having URTI in mud wall house 4.58 compared cement 4.58 (AOR (95%CI) (1.74, 12.03)]. But it is lower than the studies conducted in Iran (2007) and Kenya (2013); which indicated that all clients seen under health institutions had from mud type wall [24,25]. This may be due to the mud is appropriate for reservoir of microorganisms, difficult to clean so that respiratory tract infection simple transmitted from one to other through mucous membranes. In this study, the odds having URTI in rental house 4.91 to non-rental house 5.1 [AOR (95%CI) (1.82, 14.6)]. This is higher with the study conducted in Pakistan (1999) and Kenya (2002) [21,26]. Which revealed rental house were independent predictors of upper respiratory tract infection. This difference by house could be explained that most of the people living in the area were low economic status; lack of basic sanitation may not focus on the cleaning of room regularly and proper ventilation because of unstable house. This study showed that the odds of URTI in large family size 5.3 compared with small 5.3 [AOR (95%CI) (2.3, 12.1)]. Similar observations are also shown in the studies conducted in Atlanta (2015), Pakistan (1999) and Malaysian family size were significantly associated (1995), [21,27,28]. This may be due to overcrowding is implicated risk factor for acquiring of upper respiratory tract infection exposure with infected individuals day and night time in a single room and spend long.

## Conclusion

Based on the findings of the current study, it can be concluded that the majority of respondents, 52.6% identified as having upper respiratory tract infection and most of them were acquired 1-2 times per year. It was found that most of rural residents were acquired upper respiratory tract infection. Civil servant father children's was statistically associated with upper respiratory tract infection. Immunization is one prevention measure of upper respiratory tract infection. Overcrowding were risk factor of upper respiratory tract infection.

### What is known about this topic

- Unidentified respiratory infections are common cause of illness in under-five;
- Overcrowding, malnutrition are risk factors of respiratory problems.

### What this study adds

- More than half of the respondent identified as having upper respiratory tract infection and most of them were acquired 1-2 times per year;
- Civil servant father children's was statistically associated with upper respiratory tract infection;
- Immunization is one prevention measure of upper respiratory tract infection.
